# The Reevaluation of Subgingival Calculus: A Narrative Review

**DOI:** 10.3390/dj13060257

**Published:** 2025-06-09

**Authors:** Stephen K. Harrel, Atsutoshi Yoshimura, Charles M. Cobb

**Affiliations:** 1 Department of Periodontics, Texas A&M College of Dentistry, Dallas, TX 75246, USA; 2 Department of Periodontology and Endodontology, Nagasaki University Graduate School of Biomedical Sciences, 1-7-1 Sakamoto, Nagasaki 852-8588, Japan; ayoshi@nagasaki-u.ac.jp; 3 Department of Periodontics, School of Dentistry, University of Missouri-Kansas City, 650 E. 25th Street, Kansas City, MO 64108, USA; cobbc@umkc.edu

**Keywords:** periodontitis, dental calculus, inflammation, inflammasomes, pathogenesis

## Abstract

**Aim:** Despite a persistent presence in periodontitis, calculus remains a paradox. This narrative review reevaluates the role of calculus in periodontitis based on in situ, ex vivo, and in vitro studies published over the last two decades. **Review:** Results from multiple studies argue for the reconsideration of calculus as an independent risk factor in periodontitis. The results of a human study suggest that calculus contributes more to inflammation than simply serving as a substrate for biofilm accumulation. Ultrastructure studies have revealed residual calculus embedded in cementum following scaling and root planing (SRP). In vitro studies show that calculus particles can stimulate IL-1β secretion via the NLRP3 inflammasome in human and mouse phagocytes, and the crystalline structure is partially responsible for the activation. Other studies indicate that calculus particles may promote bone resorption via IL-1β induction in patients with periodontitis. Further, heat-treated calculus particles and hydroxyapatite crystals induce cell death in epithelial cell lines, suggesting that calculus plays a role in the breakdown of pocket epithelial integrity. **Conclusions:** Studies have shown that particles of microscopic calculus persist following traditional SRP. In vitro studies report that sterile and calcined calculus particles free of proteinaceous material are cytotoxic to cultured oral epithelial cells. Collectively, these studies suggest that residual microscopic calculus may be a potential risk factor for the failure of periodontal therapy.

## 1. Introduction

Dentistry has never settled on a defined role for dental calculus in the pathogenesis of periodontitis. Despite being almost universally present in periodontal disease, calculus remains a paradox. Historically, calculus was considered the primary cause of “gum disease”. However, the classic papers by Löe et al. [1] and Theilade et al. [2], demonstrating the role of bacteria as the primary etiologic agents in gingival inflammation, initiated the era of “plaque hypotheses”, i.e., the nonspecific and specific plaque hypotheses, the ecological plaque hypothesis, and the keystone microbe and dysbiotic biofilm hypothesis [3]. Nevertheless, there is a developing body of in situ, in vitro, and ex vivo evidence indicating that subgingival calculus, even if microscopic [4,5,6,7] or sterilized, may have a role beyond that of a substrate for biofilms in the etiology, progression, and recurrence of periodontitis [4,5,6,7,8,9,10,11,12,13,14,15,16,17,18,19,20].

Notwithstanding the evolving body of new evidence, there remains the question of why older clinical studies report that incomplete calculus removal was not associated with attachment loss [21] or did not impact clinical outcomes following access flap surgery [22,23]. Yet other studies acknowledge that subgingival calculus may contribute to the chronicity of periodontal disease, but do so without a complete understanding of the exact role and/or mechanisms involved [24,25,26]. The results of these studies, and others, prompted Robertson [27] to explain this seeming paradox by hypothesizing that there existed a threshold of residual calculus, under which the host response was tilted towards healing, and above which the response was inflammation and progression of disease.

As with much of clinical science, the continued evolution of evidence and refinement of technology often demands a re-evaluation of concepts and historical observations. As noted by Abt et al. [28], when confronted with numerous clinical studies that reach similar or dissimilar conclusions, how does one judge the importance of such studies? If similar studies reach contradictory conclusions, such as residual calculus does or does not contribute to continued periodontal inflammation or contribute to failure of periodontal therapy, how should this be interpreted? Clinical studies often use analogous designs and have similar limitations, but reach dissimilar conclusions. A common weak point in clinical studies in dentistry is that many are underpowered with regard to the size of the study population. The clinician must be aware of comorbid conditions, confounding factors, and conflation in such studies. And in relation to residual calculus, the clinician must be aware, when assessing the success of subgingival non-surgical therapy, that surrogate measures, e.g., smooth root surface, short-term lack of bleeding on probing, statistical gain in clinical attachment, reduction in probing depth, etc., do not necessarily equate to long-term success or clinical significance.

Taking into consideration the apparent enigma of subgingival calculus, the following narrative review reevaluates the role of calculus in the persistence and progression of periodontitis based on in situ, ex vivo, and in vitro studies published over the last two decades.

## 2. Review

An in situ human study by Wilson et al. [20] reported that 70% of inflamed sites in the soft tissue pocket wall were associated with distinct areas of calculus that were only visible with endoscopic magnification. The remaining 30% of inflamed sites were associated with biofilm alone. This observation suggests that calculus contributes more to inflammation than simply serving as a substrate for biofilm accumulation. Published research from multiple research groups, when viewed collectively, argues for a reconsideration of calculus as a potential independent risk factor and pathogenic agent in periodontitis [4,5,6,7,8,9,10,11,12,13,14,15,16,17,18,19,20]. In the natural history of periodontitis in Sri Lankan tea laborers, dental calculus showed a significantly stronger association with disease initiation and progression than dental plaque [21,25]. The removal of calculus showed a direct correlation with improvement in periodontal health, whereas the removal of bacterial plaque alone did not show such a correlation [29]. The percentages of residual calculus after subgingival scaling and root planing were highly correlated with pocket depth [30].

If, indeed, calculus is an independent etiologic factor, then not removing all calculus, and focusing only on bacterial agents, will result in the undertreatment of chronic periodontitis and likely contribute to recurrence of active disease.

### 2.1. Microscopic Calculus

The periodontal literature is replete with reports of residual subgingival calculus following traditional scaling and root planing (SRP). Additionally, recent studies have revealed the presence of areas of microscopic calculus following SRP [4,7]. The deposits of microscopic calculus were first noted while using a high-magnification surgical videoscope during minimally invasive surgery (VMIS) and were termed microislands of calculus [7]. The use of enamel matrix derivative (EMD) is a routine part of VMIS, and the use of 24% neutral ethylenediaminetetraacetic acid (EDTA) is a step in the use of EMD [31]. It was noted that, when EDTA was burnished on the root surface, the microislands of calculus were removed [7]. In order to show that microislands of calculus were not part of a smear layer, a study utilizing a laser wavelength that fluoresced calculus confirmed that microislands of calculus were routinely present after SRP. The microislands were, in fact, calculus, and not part of a smear layer. Further, EDTA, when burnished on the root surface, removed the majority of post-SRP residual calculus [7].

The presence of microislands of calculus correlated with the small, isolated areas of calculus shown in the Wilson et al. [20] study that linked calculus with inflammation of human periodontal lesions. At a clinical level, the microislands of calculus observed during surgical procedures with the high-magnification videoscope closely resembled the small areas of calculus observed non-surgically with an endoscope in the Wilson et al. [20] study. It is not possible with clinical observation alone to confirm that the calculus observed by Wilson et al. [20] to be associated with inflammation is the same as the microislands of calculus remaining after meticulous surgical root debridement. However, a clinical study that would confirm that microislands of calculus cause inflammation would be difficult. A human clinical study where microislands of calculus are identified and then purposely left in place to determine if they are in fact a risk factor for periodontal inflammation would violate the Declaration of Helsinki, and the study would be unethical. Because of this ethical paradox, the close resemblance of the particles of calculus, coupled with recent cell culture evidence, may be the best available evidence that can be obtained ethically.

Cell culture studies have demonstrated that sterile calculus particles can cause death of epithelial cells [17,19]. Taken together, the human study and the cell culture studies indicate that residual microislands of calculus may function as a promoter of continuing inflammation and cell death, thereby meeting the requirements of an independent risk factor for the failure of periodontal therapy. In light of these findings, further studies were performed to verify the presence of microislands of calculus after SRP and to further characterize the action of EDTA on microislands of calculus.

Cobb and Sottosanti [9], using SEM, demonstrated the presence of fractured calculus embedded in cementum following SRP. Fractured calculus and microislands of calculus appear to describe the same phenomenon. The characteristics of microislands of calculus before and after the use of EDTA were studied using SEM and Energy-Dispersive X-ray Spectroscopy [6]. This study confirmed that microislands of calculus remain after SRP, with calculus coverage approaching 50% of the test root surface, and that burnishing with EDTA removed practically all residual microislands of calculus. Further, EDTA appears to denature and alter characteristics of the biofilm, polymeric matrix, and mineral composition of calculus remnants [4,6]. A recent study [4] reports the maximum effect of EDTA can be achieved at one minute of burnishing and confirmed the severe alterations in character of calculus remnants.

Despite the short-term favorable response following SRP, the periodontal literature has been consistent in noting the presence of residual subgingival calculus associated with inflammation [30]. This observation is particularly true for pockets ≥ 5 mm and is likely responsible, at least in part, for the failure of therapy, as well as the recurrence and progression of disease [30]. Research has demonstrated that burnishing of the SRP-treated root surface with EDTA or SRP using a periodontal endoscope will remove most of the remaining microislands of calculus [4,32]. The adjunctive use of EDTA with SRP for the removal of microscopic calculus is supported by publications from Nagasaki University (Japan) that demonstrate the toxicity of calculus particles to cells in vitro.

### 2.2. In Vitro Experimental Studies

The composition of dental calculus is 70–80% inorganic, with the remainder comprised of organic components [33]. Prior to the formation of dental calculus, organic substances adhere to tooth surfaces from several sources, including oral bacteria, saliva, serum, leukocytes, epithelial cells, and food remnants [34]. Among them, bacteria are thought to play a major role in the formation of dental calculus. Mineralization occurs in the form of extracellular or intracellular calcifications [35]. Bacteria have a large surface area to volume ratio, and their surfaces are generally anionically charged, causing extra bacterial calcification by providing interfaces for the sorption of metal cations from the surrounding aqueous environment [36]. Intracellular calcification appears to be promoted by phosphoproteins in dead bacteria [37]. Bacterial elements contained in calculus are recognized by host innate immune receptors such as Toll-like receptors (TLRs) [38]. Stimulation through these innate immune receptors triggers the transcription of pro-IL-1β via the activation of nuclear factor-κB. Pro-interleukin (IL)-1β is biologically inactive and is cleaved to its active form by a proteinase called caspase-1 [39]. Active caspase-1 can be generated from pro-caspase-1 by autocatalysis upon inflammasome assembly. Inflammasomes consist of a sensor molecule, such as nucleotide-binding oligomerization domain-like receptor (NLR), apoptosis speck-like protein containing a caspase-recruitment and activation domain (ASC), and pro-caspase-1 [40].

Several sensor molecules form inflammasomes, including NLRP1, NLRP3, NLRC4, and AIM2, with the NLRP3 inflammasome being the most studied. Assembly of the NLRP3 inflammasome can be triggered by the cellular uptake of crystalline particles. Consistent with these studies, Montenegro Raudales et al. [12] found that pulverized dental calculus induces IL-1β secretion in both human polymorphonuclear leukocytes and peripheral blood mononuclear cells. Dental calculus also induces IL-1β release in macrophages from wild-type mice, but not in macrophages from NLRP3-deficient or ASC-deficient mice, indicating the involvement of NLRP3 and ASC. IL-1β induction was inhibited by the LPS inhibitor, polymyxin B, suggesting that LPS plays a major role in the induction of pro-IL-1β transcription. When calculus was baked at 250 °C for 1 h, it failed to induce pro-IL-1β transcription. However, it induced IL-1β secretion in lipid A-primed cells, indicating that the crystalline structure of calculus induces inflammasome activation. Furthermore, hydroxyapatite crystals, a component of dental calculus, induce IL-1β release in mouse macrophages primed with lipid A [12]. These results indicate that dental calculus stimulates IL-1β secretion via the NLRP3 inflammasome in human and mouse phagocytes, and that the crystalline structure is at least partially responsible for the activation of the NLRP3 inflammasome.

IL-1β levels in gingival tissues and gingival crevicular fluids correlate with the inflammatory status of periodontal disease, suggesting that IL-1β induced by dental calculus contributes to the inflammatory responses in periodontal tissues [41]. IL-1 receptor type 1 is expressed in almost all cell types in periodontal tissue [42]. Through its interaction with its receptor, IL-1β modulates the metabolism of connective tissue and gingival epithelial cells, and induces proinflammatory cytokines and chemokines, which in turn induce the migration and activation of leukocytes to promote inflammation [43,44]. Furthermore, Mae et al. [15] found that culture supernatants from wild-type (WT) mouse macrophages stimulated with calculus, which contain IL-1β, accelerated osteoclastogenesis in RANKL-primed mouse bone marrow macrophages (BMMs). These results suggest that calculus may promote alveolar bone resorption via IL-1β induction in patients with periodontitis.

Similar to IL-1β processing, NLRP3 inflammasome activation was reported to lead to the processing of gasdermin D by inflammatory caspases. After cleavage, N-terminal fragments of gasdermin D assemble into pores in the cell membrane, causing a cell death, pyroptosis [45]. On the other hand, Du et al. [46] have recently demonstrated that *S*-palmitoylation of gasdermin D is required for pore formation by both the N-terminal fragment of gasdermin D and intact gasdermin D. Ziauddin et al. [14] exposed HSC-2 human oral squamous carcinoma cells and HOMK107 human primary oral epithelial cells to dental calculus, which induced cell death, as determined by LDH release and propidium iodide staining. Heat-treated dental calculus and its component hydroxyapatite crystals also induced cell death in both HSC-2 and HOMK107 cells, indicating an essential role of crystal particles in cell death. It is worth noting that these oral epithelial cells are less sensitive to microorganisms than inflammatory cells, and are therefore resistant to cell death [19]. This is one of the reasons why dental calculus is considered a possible important etiological factor.

In permeability assays, dental calculus attenuated the barrier function of HSC-2 cell monolayers. Ziauddin et al. [19] also compared the effects of dental calculus and freeze-dried periodontopathic bacteria on oral epithelial cells. In permeability assays, dental calculus, but not freeze-dried bacteria, attenuated the barrier function of HSC-2 cell monolayers, suggesting that dental calculus plays a critical role in the breakdown of crevicular/pocket epithelium integrity. However, these findings were based on in vitro experiments and need to be verified in vivo. Schroeder et al. [47] found that ligatures and a soft diet for 36 months resulted in the formation of partly mineralized subgingival plaque, attachment loss, and apparent net loss of alveolar bone in beagle dogs. This finding suggests that partly mineralized subgingival plaque containing small crystalline particles may contribute to the conversion from stable established gingivitis to destructive periodontitis. Experimental animal models in which calculus particles are administered into the gingival sulcus may reveal the role of calculus in the breakdown of the barrier function of the crevicular/pocket epithelium.

## 3. Discussion

There is little doubt that scaling and root planing are often effective treatments for Stage I–III periodontitis, resulting in reductions in clinical inflammation and pocket probing depth. Yet, multiple studies report that, following SRP, a significant percentage of treated teeth will exhibit residual subgingival biofilm and calculus. Not surprisingly, a higher percentage of deep periodontal pockets (≥6 mm) will exhibit more residual calculus than those with a shallower depth [30,48,49,50,51].

And yet, the cemento-enamel junction is a common site for finding deposits of residual calculus [52]. Even in vitro studies using extracted teeth or calculus-laden root chips report that removing all calculus by SRP is seldom achieved [4,6,53]. Obviously, the removal of subgingival calculus presents a challenge to the best of clinicians and raises the question of how much residual calculus is too much, i.e., is there truly a threshold, as suggested by Robertson [27]?

The fact remains that no study has ever determined if, in fact, a threshold exists for subgingival calculus, below which there is no inflammation or disease progression. Simply put, is there a point where subgingival calculus becomes compatible with periodontal health? There is overwhelming evidence that subgingival calculus, residual or otherwise, is related to inflammation and disease progression [26,34,54]. Given the evidence, it seems reasonable to err on the side of caution and remove all subgingival calculus, or at least neutralize, in some manner, the potential pathologic impact of any residuum.

There remains the conundrum created by reports that incomplete calculus removal was not associated with attachment loss [21] or did not impact clinical outcomes following access flap surgery [22,23]. Given the variability in biological systems, it is highly probable that the composition of calculus mirrors that variability. There can be measurable differences in composition, with the end-product still referred to clinically as dental calculus. For example, the inorganic constituent of subgingival calculus can vary between 58% [55] and 75–80% [33,56,57] in the form of calcium hydroxyapatite, brushite, whitlockite, and octacalcium phosphate [58,59,60,61]. The organic material composition of calculus can vary from 6–28% of total dry weight [62,63], consisting primarily of bacteria and their extracellular polymeric matrices. Owing to the marked roughness of its outer surface, dental calculus almost always harbors a living, non-mineralized biofilm [34]. A variety of viable and nonviable bacteria are progressively mineralized from extracellular substrates to the whole bacteria. These small crystalline particles in partly mineralized biofilms can be incorporated into phagocytes and epithelial cells, triggering the assembly of the NLRP3 inflammasome [12]. However, if the accumulation of organic material and bacteria is prevented, calculus nanoparticles will not develop. The amount and roughness of calculus remaining on the root surface after instrumentation would contribute to biofilm formation and the subsequent generation of small crystalline particles. Hence, the relatively smooth surface of the residual calculus after instrumentation and good oral hygiene status may partially explain the lack of association between incomplete calculus removal and attachment loss, as described above. Nevertheless, it is ideal to remove the calculus completely, because there is no measurable threshold. It is interesting to note that the use of EDTA on residual microislands of calculus alters their mineral composition [6]. Future research may show such EDTA-induced change to have a positive impact on the pathogenic potential of residual calculus.

The attachment of epithelium to any surface, including a biologic substrate such as calculus, is likely regulated by other factors. For example, physicochemical surface properties play a major role in the cell adhesion process. In vitro cell adhesion to any surface, including extracellular matrix, can be manipulated by the composition and physical properties of the substrate, e.g., surface porosity, chemistry, and charge [64,65,66,67]. It is well established that fibroblast and epithelial cell lines can adhere to silica-based materials, phosphate-based materials, various bioactive glasses, plastics, dental implant surface materials, tooth enamel, and root cementum [64,65,66,67,68]. In the case of epithelial cells, electron microscopic images show their attachment to be similar for all substrates, both biologic and synthetic [69]. Thus, it is not surprising that junctional epithelium has been observed in close approximation, and possibly attached, to the surface of subgingival calculus [70,71]. However, it should be noted that such observations are extremely rare in the research literature. The authors of this review suggest that this phenomenon is most likely to occur on older calculus in which the majority of microbes are calcified [55], and/or on areas devoid of surface biofilm. Indeed, Bercy and Frank [72] reported finding areas of subgingival calculus that were devoid of bacteria. Listgarten and Ellegaard [70] were the first to show that, under specific conditions, notably the lack of surface biofilm, it was possible for junctional epithelium to attach to subgingival calculus. However, they suggested that the use of antibacterial rinses, such as chlorhexidine, may lower the toxicity of calculus to a level that allows the adherence of epithelial cells. Collectively, these factors must be balanced with the results of earlier publications [21,22,23] that appear to challenge the idea that calculus is a noxious accretion associated with periodontal disease.

Lastly, the results of earlier studies [21,22,23] failed to address such questions as the following: How long does epithelial adherence to a calculus surface last? And, should it occur, does it last long enough to be clinically significant, or is it simply an aberrant biologic phenomenon? Could such an adherence account for the results of the studies, or do such studies imply that it is not necessary to remove all calculus? Current evidence would suggest that, if such an adherence occurs at all, it is likely to be short-lived, and the formation of subgingival calculus may be more complex than previously thought [12,13,14,15,17,19,20,73,74,75,76].

In summary, while there is no question of the validity that periodontitis is primarily a biofilm-initiated disease, new evidence points to the possibility that calculus plays a role, beyond a roughened surface that contributes to the formation and retention of biofilm. While further research is progressing to more fully explore the potential role of calculus as an independent risk factor for periodontitis, the data gathered to date points in this direction. If calculus is an independent risk factor for periodontitis, it is in addition to the biofilm-induced phenomenon, and not a replacement or negation of the biofilm pathway to periodontal inflammation.

## 4. Conclusions

In this narrative review, we have summarized two sets of independent data; one from the U.S., showing that particles of microscopic calculus (microislands) are routinely present following traditional SRP; and the other from Nagasaki University (Japan), showing that sterile or even calcined calculus particles free of proteinaceous material are cytotoxic to cultured oral epithelial cells. Taken together, these studies indicate that residual calculus, if it has the potential to re-form small crystalline particles, has a high probability of being a risk factor for the failure of periodontal therapy.

Our findings do not challenge the concept that periodontal inflammation is centered on the oral microbiome and the host inflammatory response. Our studies look instead at calculus as a potential risk factor for continued inflammation and attachment loss in inadequately treated patients.

Long-term success in the treatment of periodontitis requires the elimination of all factors that initiate and promote inflammation [25,76,77,78]. The attainment of this goal requires the removal of subgingival plaque and calculus, the establishment of oral hygiene, supportive periodontal maintenance at appropriate intervals, and continual re-evaluation of periodontal status. Clinicians proficient in the treatment of periodontitis know the challenges involved to achieve long-lasting success.
